# Taking microvascular training through the elevator: institutional experience in structured microsurgical training in India

**DOI:** 10.1186/s40902-026-00507-x

**Published:** 2026-05-27

**Authors:** Navneet Kaur, Vineet Kumar, Mayur Mantri, Ameya Bindu, Saumya Mathews, Kunal Mokhale, Dushyan Jaiswal, Vinay Kant Shankhdhar

**Affiliations:** 1 Department of plastic & reconstructive surgery, Tata Memorial Hospital, Mumbai, India; 2 Tata Memorial Hospital, Mumbai, Homi Bhabha National Institute, Mumbai, India

**Keywords:** Microsurgery, Structured training, Head and neck reconstruction, Breast reconstruction, Free flap surgery, Competency-based training

## Abstract

**Background:**

Microsurgery is an essential skill in plastic surgery, particularly for oncological reconstructions. The traditional training pathway in India spans approximately 12 years, encompassing medical school, general surgery residency and plastic surgery specialization. Given the projected 12.8% rise in cancer incidence by 2025 in India, there’s an urgent need to expedite microsurgical training without compromising patient care quality.

**Methods:**

At a tertiary cancer institute, a structured four-phase microsurgical training program was implemented from April 2022 to December 2023 for second-year plastic surgery residents. The program incorporated Peyton’s four-step teaching approach—demonstration, deconstruction, comprehension, and performance—and utilized simulation-based training with latex gloves, silicon tubes, and live rat models. Progression to subsequent phases required achieving a minimum score of 8/10 on the Microsurgical Anastomosis Rating Scale (MARS10). Residents advanced from assisting in flap harvests to independently performing complete microsurgical procedures.

**Results:**

Over the study period, 1,520 free flap surgeries were performed in our institute. Residents individually conducted between 73 and 79 flap harvests and 18 to 22 microvascular anastomosis. The flap survival rate among resident-performed surgeries was 94.73%, close to the institute’s overall rate of 95.53%. According to statistical analysis, there was no significant difference (*p* = 0.812), indicating the training model’s effectiveness.

**Conclusion:**

The structured training model effectively reduced the microsurgical learning curve, maintaining high standards of patient care. By integrating simulation-based practice and objective assessment, the program offers a replicable framework for accelerating microsurgical competency among plastic surgery residents.

**Supplementary Information:**

The online version contains supplementary material available at 10.1186/s40902-026-00507-x.

## Introduction

The field of microsurgery has seen remarkable expansion over the last few decades, offering a vast range of applications. These include, but are not limited to, oncoreconstruction, breast reconstruction, limb salvage, lymphedema treatment and gender affirmation surgery [1]. Microsurgery has become an indispensable skill set, significantly enhancing the reconstructive capabilities of plastic surgeons. The learning curve for the same is long as acquiring microsurgery skill set demands precise hand-eye coordination and consistent practice, relying on a distinct set of principles that sets it apart from conventional surgical skills. Villanueva et al. used advanced statistical modeling to characterize the multifaceted microsurgical learning curve and objectively delineate distinct phases of skill progression based on time, error rates, and performance indices [2].

Free flap transfer using microvascular anastomosis technique is the standard of reconstruction in Head and Neck and breast cancers (reconstructive ladder). According to the National Cancer Registry Programme (NCRP) of India, an estimated 1.46 million new cancer cases were diagnosed in 2022, corresponding to a crude incidence rate of 100.4 per 100,000 population, and this burden is projected to increase by approximately 12.8% by 2025 [3]. To meet the increased demand, expediting microvascular training in young plastic surgeons/residents is crucial.

Over the past decade, surgical training has been transformed by two groundbreaking concepts, now integrated across nearly all surgical fields, including microsurgery. These innovations are the objective evaluation of surgical skills and the cultivation of these skills within simulation laboratory environments [4]. The microsurgical training in residencies or fellowships demands a systematised, well-structured, and proven teaching methodology. Historically, the teaching of surgical skills has followed the “see one-do one” method, where an instructor demonstrates and explains a procedure, after which students attempt to perform it themselves. However, Walker and Peyton have introduced a more contemporary approach to teaching procedural skills. This method, known as Peyton’s four-step approach, involves a systematic process that includes demonstration, deconstruction, comprehension, and performance. This structured approach aids in more effectively acquiring and mastering procedural skills [5]. In this context, skill refers specifically to technical microsurgical competence in free-flap reconstruction, including flap harvest, atraumatic tissue handling, microvascular vessel preparation, execution of end-to-end microvascular anastomosis, and completion of the reconstructive procedure under structured supervision. However, it’s objectivity as a skill-acquisition tool remains limited by the inherent need for a trainer to be present and engaged with the operator. Contemporary surgical training increasingly incorporates objective assessment frameworks such as OSATS and Global Rating Scales (GRS), which enable standardized evaluation of technical competence. These tools complement Peyton’s method by addressing its limitations in objectivity and assessment, rather than replacing its role in structured skill acquisition. Dr. Hallock posits that these four P’s—perfectionist, pragmatist, persistent, and paranoid—are interwoven and inseparable traits that collectively define the essence of a microsurgeon [6].

Our approach introduces a structured educational model that combines laboratory-based microsurgical training with progressive, closely supervised clinical operative exposure, and is associated with favorable microsurgical outcomes while maintaining the quality and safety of surgical care.

## Materials and methods

This study involves microsurgical and free flaps transfer training of plastic surgery residents for a period from April 2022 to December 2023 in a tertiary cancer institute with the majority of cases in Head and Neck cancers and Breast cancers, focusing mainly on now second-year residents.All second-year plastic surgery residents undergoing structured microsurgical training during the study period were included in the analysis. No additional inclusion or exclusion criteria were applied, as the training model was implemented uniformly across all residents during this period. The training process is divided into four phases, with six months each, and each phase is subdivided into two three-month periods. This is retrospective institutional audit and observational evaluation of the training program. Hence Institutional review board approval was not needed in this case.

The outcomes were compared with both our overall institute results and international standards specifically the flap success. The phases involved were. 

### Phase 1

Participating as the second assistant in the harvest for three months, each resident contributed to thirty free flap harvests, primarily involving the Free Fibula Osseocutaneous flap, Free Anterolateral Flap, and Free Radial Artery Forearm flap. 

Over the next three months, the residents transitioned to serving as first assistants in flap harvests. During the assistant phase, residents participated as first assistants with predefined responsibilities including tissue handling, exposure, retraction, instrument exchange, suction and hemostasis under direct consultant supervision. This assistant role was implemented as a structured preparatory step for subsequent supervised operative responsibility and was not used as a measured outcome variable in the present study. Concurrently, they began assisting in microvascular anastomosis procedures. 

### Phase 2

In the subsequent stage, residents commenced their hands-on practice of microvascular anastomosis in the laboratory. This initiation involved honing suturing techniques on latex gloves and progressing to weekly practice sessions on rats. We have further strengthened the animal ethics that our experimental microsurgical training follows international standards recommended by the European Society for Surgical Research (ESSR) and the International Society for Experimental Microsurgery (ISEM) (Tolba et al) [7]. Simultaneously, within the operating theatre during this phase, they incrementally undertook flap harvests in a step-wise manner under supervision. Over the ensuing three months, residents accomplished complete flap harvests. Concurrently, they continued their roles in assisting with microvascular anastomosis procedures. Residents were permitted to progress to the subsequent phase of the program only after achieving a minimum MARS10 score of 8/10 in live rat models on three consecutive assessments. 

### Phase 3

This stage commenced with residents performing the second vein anastomosis in head and neck cases. Throughout this period, residents played a supporting role in osteotomies and the flap’s inset. By the conclusion of this phase, they gained the ability to independently harvest the flap and, while still assisting and learning osteotomy and inset techniques, they were supervised in microvascular anastomosis. 

### Phase 4

During the initial segment of this phase, residents demonstrated competent performance in executing complete cases, encompassing harvest, osteotomy, inset, and microvascular anastomosis, all under supervision. Subsequently, they were deployed to peripheral centres, where they performed supervised cases.

## Results

The total number of free flaps in the institute from April 2022 to December 2023 was1520. Out of these, free fibula flaps were 494, free anterolateral thigh flaps were 599, free radial artery forearm flaps were 191 and deep inferior epigastric artery flaps were 147. Clinical outcomes were assessed using flap survival rate as the primary endpoint and re-exploration and complication rates as secondary endpoints. The overall flap survival rate, excluding the study cases from the institute, was 95.53%. The flap survival rate of this study was 94.73%. The results of the two-sample proportion test showed a very small, non-significant difference between the overall flap survival rate (95.53%) and the flap survival rate observed in this study (94.73%), with Z = 0.24 and *p* = 0.812. The 95% confidence interval ranged from − 0.0454 to 0.0572. The comparison between resident-performed cases and the institutional audit was conducted as an exploratory patient-safety comparison. The analysis was not designed as a formal non-inferiority or equivalence study, and no predefined non-inferiority margin was specified. Proportions were compared using a two-sample test, and confidence intervals were calculated where applicable.

The following were the results of our model (Tables 1, 2, 3 and 4).

**Table 1:** Number of free flaps harvested by each resident

Flap harvest	Resident I	Resident II	Resident III	Resident IV
FALT	42	40	41	36
FFOCF	23	25	30	29
FRAFF	8	11	8	12
DIEP	0	0	0	1
Total	73	76	79	77

**Table 2:** Number of microvascular anastomosis performed by each resident

Microvascular anastomosis	Resident I	Resident II	Resident III	Resident IV
Lab models	5	4	7	6
Rat	5	6	4	5
2^nd^ vein in a patient	10	14	12	11
Supervised	4	5	3	6
Independent	19	22	18	20

**Table 3:** Number of free flaps done by each resident

	Resident I	Resident II	Resident III	Resident IV
FFOCF	6	4	5	5
FALT	10	9	11	10
FRAFF	4	5	3	4
DIEP	0	0	0	0
Re-exploration Rate	2	1	3	2
Flap survival Rate	19/20 = 95%	17/18 = 94.4%	18/19 = 94.7%	18/19 = 94.7%

**Table 4:** Re-exploration rate

Resident	Total number of free flaps done	Re-explorations (*n*)	Re-exploration rate (%)
Resident I	20	2	10
Resident II	18	1	5.6
Resident III	19	3	15.8
Resident IV	19	2	10.5
Overall	76	8	10.5

## Discussion

Microsurgery demands the technical expertise and skills which are earned with practice and experience. In India, the curriculum is such that first, the medical student needs to finish five and a half years of a bachelor’s in medicine and a bachelor’s in surgery. After that, there are three years of residency in general surgery. Then they can enter the plastic surgery residency of three years. Still, this is not enough for the microsurgery required for oncological reconstruction. Hence, one or two-year fellowships are mandatory to get hands-on on with free flaps. This means it takes a minimum of 12 years to train for microsurgery. But the demand for head and neck reconstruction is huge in India, and there is a need to shorten the learning curve with adequate exposure and not only without compromising the quality of care to the patients but also the quality of training to the students. Our institute is the largest academic institute in India for head and neck reconstruction, with around 1500 free flaps in a year. Following implementation of our structured program, the institutional training pathway required for residents to reach supervised competence in free-flap reconstruction was shortened from the traditionally followed 12-year pathway to a 10-year pathway at our institute, while maintaining a flap survival rate of 94.7% in resident-performed cases. In the Indian training pathway, the “12 years” refers to the cumulative duration of formal surgical training, including undergraduate education, general surgery residency, plastic surgery residency, and often additional microsurgery fellowship. This can be seen as programmatic restructuring of the institutional training pathway and earlier supervised entrustment, rather than a quantified or validated individual learning-curve reduction. The training model includes systemic and step-wise training in onco-reconstruction, namely flap planning, harvesting, osteotomy, inset and microvascular anastomosis. With over 1500 free flaps performed over two years, the surgical steps are uniform and standardized, which makes it easier to understand and acquire later.

Implementing the Walker and Peyton approach, residents underwent intensive training. As all residents had prior training in general surgery, the initiation of training involved acquiring a fundamental understanding of procedures such as flap harvest through demonstrations in the operating theatre. Subsequently, they progressed to the role of the first assistant for these procedures performing tissue handling, instrument exchange and hemostasis to support the primary surgeon, providing key insight into training effectiveness. Supervised flap harvest commenced once they demonstrated a strong grasp of the concepts verified through active assistance during the procedure and assessment of their responses and technical understanding. Each resident harvested 73–77 flaps in their first year of training, which was initially supervised and followed by independent harvest. Concurrently, residents actively participated in the designing, planning, and execution of the reconstruction process.

According to DrGeoffrey G. Hallockteaching must serve as a foundational prerequisite for effectively spreading the gospel, starting with ourselves through the disciplined application of “repetition, repetition, and repetition” within the context of our animal laboratory [8]. Simultaneously, Residents started practicing in the microvascular practice lab. Various training methods for microvascular anastomosis have been introduced, including models using gauze, silicon tubes, cryopreserved rat vessels, or living rats. In these models, fidelity and accessibility or repeatability are inconsistent. Training followed a stepwise progression from low- to high-fidelity models. Residents began with basic suturing on latex gloves, followed by silicone tubes and cryopreserved vessels, which provide cost-effective, reproducible platforms for acquiring fundamental microsurgical skills. Progressions to a living animal model was undertaken after achieving basic proficiency, as in vivo models offer higher fidelity with dynamic factors such as vessel characteristics and bleeding, but are limited by cost, ethical considerations, and accessibility [9]. Using a living animal model, residents have the opportunity to learn not only anastomotic techniques but also haemostatic control [10].

Therefore, role of rat models in this study was to complement earlier simulation stages rather than replace them, ensuring a balanced, safe, and resource-conscious training pathway. Their skills were evaluated at various steps using an objective scoring system, i.e., MARS10. Several evaluation tools are commonly used in microsurgical courses, with the most prevalent being the Global Rating Scale (GRS), which relies on direct observation guided by objective criteria. The MARS10 scale, on the other hand, focuses solely on assessing the outcome of a completed end-to-end arterial anastomosis, representing just one component of the full GRS evaluation. Assessment in this study was performed using MARS10 as a measure of the final anastomotic outcome rather than intraoperative technical performance. The 10-point Microsurgical Anastomosis Rating Scale (MARS10) is a valid and reliable tool for assessing end-to-end arterial anastomosis [11]. Assessment in this study was performed using MARS10 as a measure of the final anastomotic outcome rather than intraoperative technical performance. Although evaluator blinding, motion-based objective metrics and automated performance tracking were not available, assessment bias was mitigated by the use of a standardized scoring rubric, predefined progression criteria and repeated evaluations under uniform laboratory conditions. All assessments were performed by trained supervising faculty involved in microsurgical training. Residents were allowed to go on to the next phase only after obtaining a minimum of 8/10 MARS scoring in live rat models 3 consecutive times. Hirche et al. demonstrate that free flaps—often regarded as the pinnacle of microsurgical practice—can be safely utilized as a teaching procedure for residents when performed under standardized conditions [12]. Based on the observed flap survival rates (94.73% in resident-performed cases versus 95.53% in institutional non-study cases), the clinical outcomes of the structured training pathway appear comparable to those observed in routine institutional practice. The assessment of general and microsurgery-specific outcome measures showed no significant distinctions. The overall flap survival rate was 95.53%, whereas the flap survival rate in this study was 94.73%. Currently, reported success rates for free flap reconstruction in head and neck cancer range from 91% to 99%, reflecting the procedure’s reliability and effectiveness in experienced hands [13–15]. The success rates for free flap oncoreconstruction in head and neck of our residents are comparable to international success rates. No variations in major complications were detected, including total flap loss, anastomotic thrombosis, partial necrosis (> 30%), deep surgical site infections or hematoma that needed surgical drainage. The minor complications like Small wound dehiscence, minor partial necrosis, seroma/hematoma managed by aspiration, superficial infection were also comparable. The traditionally perceived long learning curve in microsurgery can be significantly reduced. Structured training and proper supervision play a key role in achieving this. The current study possesses certain limitations that warrant discussion. It is designed retrospectively, introducing the possibility of bias in the allocation of patients and procedural characteristics and inclusion of only four residents limits statistical power and generalizability. The generalisability of this training model depends on institutional case volume, availability of experienced faculty for close supervision, and access to structured simulation and laboratory facilities. A sufficiently high and diverse oncologic reconstructive workload is required to allow stepwise entrustment of residents. Therefore, implementation of this model may be challenging in low-volume or resource-limited centres, and local adaptation based on operative volume, supervision capacity and infrastructure is necessary before wider adoption.

## Conclusion

This structured model highlights the educational value of competency-based, phase-wise entrustment while maintaining patient safety during high-risk oncologic reconstruction. Future studies should focus on prospective and multicentre validation using standardized competency metrics and risk-adjusted clinical outcomes to strengthen the evidence and generalisability of this training pathway.

## Supplementary Information

Supplementary Material 1.

## Data Availability

No datasets were generated or analysed during the current study.

## Abbreviations

MARS10: Microsurgical Anastomosis Rating Scale (10–point version)
FFOCF: Free Fibula Osseocutaneous Flap
FALT: Free Anterolateral Thigh Flap
FRAFF: Free Radial Artery Forearm Flap
DIEP: Deep Inferior Epigastric Artery Perforator Flap
GRS: Global Rating Scale
