# Supplementary material for: Taking microvascular training through the elevator: institutional experience in structured microsurgical training in India
Source: Maxillofac Plast Reconstr Surg. 2026 May 27;48(1):13. doi: 10.1186/s40902-026-00507-x (PMC13216362; doi:10.1186/s40902-026-00507-x)
Supplement: Supplementary file 1 — Supplementary Material 1. [file 40902_2026_507_MOESM1_ESM.docx]

‘Human Ethics and Consent to Participate declarations: not applicable’.

The study was conducted as per good clinical practices and in accordance with the declaration of Helsinki.

As the study is retrospective audit and does not involve any patient participation, ethics committee approval was not necessary in this case, hence not taken.

There was no funding used
